# The lack of aspirin resistance in patients with coronary artery disease

**DOI:** 10.1186/s12967-016-0827-7

**Published:** 2016-03-15

**Authors:** Nóra Homoródi, Emese G. Kovács, Sarolta Leé, Éva Katona, Amir H. Shemirani, Gizella Haramura, László Balogh, Zsuzsanna Bereczky, Gabriella Szőke, Hajna Péterfy, Róbert G. Kiss, István Édes, László Muszbek

**Affiliations:** Institute of Cardiology and Heart Surgery, University of Debrecen, 22 Móricz Zsigmond Krt., 4032 Debrecen, Hungary; Division of Clinical Laboratory Science, Department of Laboratory Medicine, University of Debrecen, 98 Nagyerdei Krt., 4032 Debrecen, Hungary; Department of Cardiology, Military Hospital, 44 Róbert Károly Krt., 1134 Budapest, Hungary; Vascular Biology, Thrombosis and Hemostasis Research Group of the Hungarian Academy of Science, University of Debrecen, 98 Nagyerdei Krt., 4032 Debrecen, Hungary; Diagnosticum Co., Research Laboratory, 126 Attila u., 1046 Budapest, Hungary

**Keywords:** Aspirin, Coronary artery disease, Cyclooxygenase-1, Cyclooxygenase-2, Platelet, Thromboxane B_2_

## Abstract

**Background:**

Aspirin resistance established by different laboratory methods is still a debated problem. Using COX1 specific methods no aspirin resistance was detected among healthy volunteers. Here we tested the effect of chronic aspirin treatment on platelets from patients with stable coronary artery disease. The expression of COX2 mRNA in platelets and its influences on the effect of aspirin was also investigated.

**Methods:**

One hundred and forty four patients were enrolled in the study. The direct measurement of COX1 acetylation was carried out by monoclonal antibodies specific to acetylated and non-acetylated COX1 (acCOX1 and nacCOX1) using Western blotting technique. Arachidonic acid (AA) induced TXB_2_ production by platelets was measured by competitive immunoassay. AA induced platelet aggregation, ATP secretion and VerifyNow Aspirin Assay were also performed. COX2 and COX1 mRNA expression in platelets were measured in 56 patients by RT-qPCR.

**Results:**

In 138 patients only acCOX1 was detected, in the remaining six patients nacCOX1 disappeared after a compliance period. AA induced TXB_2_ production by platelets was very low in all patients including the 6 patients after compliance. AA induced platelet aggregation, secretion and with a few exceptions the VerifyNow Assay also demonstrated the effect of aspirin. Smoking, diabetes mellitus and inflammatory conditions did not influence the results. The very low amount of COX2 mRNA detected in 39 % of the investigated platelets did not influence the effect of aspirin.

**Conclusions:**

No aspirin resistance was detected among patients with stable coronary artery disease. COX2 expression in platelets did not influence the effect of aspirin.

## Background

Since the middle of the last century low dose aspirin (acetylsalicylic acid) has been effectively used in the prevention of acute atherothrombotic complications, like myocardial infarction and atherothrombotic ischemic stroke [1–5]. It prevents vascular death and nonfatal vascular events by approximately 15 and 30 %, respectively [1–3, 6]. The antithrombotic effect of aspirin is primarily due to its anti-platelet action. Aspirin as an acetylating agent acetylates the Ser529 residue of cyclooxygenase-1 (COX1), which is located in the wall of the enzyme’s active-site pocket [6–8]. Arachidonic acid (AA) released from membrane phospholipids as the result of signalization by platelet agonists, is transformed by COX1, exerting both cyclooxygenase and peroxidase activities, into cyclic endoperoxides, prostaglandin G_2_ and H_2_. The latter is the precursor of the powerful platelet activating prostaglandin derivative, thromboxane A_2_ (TXA_2_). The covalent modification of Ser529 prevents the access of AA to the active site of COX-1 and blocks TXA_2_ production by platelet throughout its lifespan.

In certain patients low dose aspirin therapy is not effective in preventing acute atherothrombotic complications, and for this reason the term “aspirin resistance” was introduced. In the PubMed browsing this term resulted in over 2000 publications. True aspirin resistance is the inability of aspirin to acetylate Ser259 residue in platelet COX1. The ineffectiveness of aspirin in protecting an individual from acute vascular events is often considered “aspirin resistance” although such cases are very likely related to the fact that aspirin inhibits effectively and selectively only one out of several pathways leading to platelet activation [9, 10]. It fails to inhibit platelet activation induced by strong agonists, like thrombin or high dose of collagen and only partially inhibits ADP induced platelet aggregation. The involvement of such stimuli in platelet activation could be prevalent in atherosclerotic vessel. In many studies non-compliance was not adequately controlled and was not separated from aspirin resistance. Further problem was the lack of laboratory tests that specifically detect the acetylation of COX1. Non-responsiveness to aspirin as measured by laboratory tests non-specific for detecting the effect of aspirin was also confused with aspirin resistance. In a recent study we developed monoclonal antibodies reacting with acetylated and non-acetylated COX1 (acCOX1 and nacCOX1, respectively) for the first time, and utilizing these antibodies a highly specific and sensitive method that detects 2.5 % of platelet nacCOX1 was developed [11]. Using this method that measures COX1 acetylation by aspirin directly, no aspirin resistance was found among 108 healthy volunteers taking 100 mg enteric-coated aspirin daily for 7 days. The same results were obtained by COX1-dependent functional tests, like AA-induced TXB_2_ formation in platelet rich plasma (PRP), AA-induced platelet aggregation and the VerifyNow Aspirin Assay [11, 12]. Practically complete inhibition of TXB_2_ formation during coagulation of whole blood was also observed in 24 healthy volunteers after 7 days of aspirin treatment [13].

However, in patients with coronary artery disease (CAD) the situation might be different from that observed in healthy volunteers. There could be conditions related to this disease, which might influence the effect of aspirin. The higher turnover of platelets and the expression/up-regulation of COX2, insensitive to low dose aspirin, have been claimed to contribute to impaired aspirin effect in diabetic patients and in inflammatory conditions [9, 14–17]. To explore the frequency of true aspirin resistance among patients with CAD 144 such patients being on long-term aspirin monotherapy were investigated for COX1 acetylation and tested for COX1 dependent functional assays. The expression of COX2 in this patient population was also investigated.

## Methods

### Subjects

Patients who had undergone percutan coronary angioplasty or coronary artery bypass grafting surgery being on aspirin monotherapy for secondary prevention were enrolled in the study. The patients were regularly admitted at the outpatient service of the Department of Cardiology, University of Debrecen, Debrecen Hungary; study participants were enrolled within a 1.5-year period. They were taking 100 mg enteric-coated aspirin daily for at least 1 month before investigation. Exclusion criteria were: acute coronary syndrome or any other major clinical event or infection in the previous 3 months, malignant disorder, hyperthyroidism, autoimmune disease and chronic renal insufficiency, intake of antiplatelet drugs other than aspirin in the previous 2 weeks, known bleeding diathesis, anemia with hemoglobin value below 100 g/L and platelet count below 100 G/L. Finally, 144 patients were eligible for being involved in the study. The Jeffrey method was used for sample size calculation as described by Brown et al. [18]. Apparently healthy controls (n = 108) recruited for the study have been characterized in a previous publication [11]. All individuals enrolled in the study gave written informed consent prior to their inclusion in the study. Ethical approval was obtained from the Ethics Committee of the Medical Faculty, University of Debrecen, Hungary and the study was performed in accordance with the ethical standards laid down in the 1964 Declaration of Helsinki and its later amendments.

### Sample preparation

Blood samples were collected into Vacutainer tubes containing 0.109 mol/L trisodium citrate (Becton–Dickinson, Franklin Lakes, NJ) after overnight fasting. Anticoagulated blood was directly used for the VerifyNow^®^ (VN) Aspirin assay (Accumetrics, San Diego, CA). Platelet rich plasma (PRP) separated by centrifugation (120*g*, 37 °C, 15 min) was used for AA induced platelet aggregation tests. Platelet depleted plasma (PDP), was obtained by two consecutive centrifugations (1500*g*, 25 °C, 20 min).

### Methods used for the detection of aspirin effect

In a previous study we developed new methods for the direct and indirect detection of COX-1 acetylation by aspirin [11]. The first method is based on two monoclonal antibodies raised against the acetylated and non-acetylated nonapeptide that corresponded to human COX-1 525-533 residues (H-Gly-Ala-Pro-Phe-Ser-Leu-Lys-Gly-Leu-OH). Using the two purified antibodies nacCOX1 and acCOX-1 could be clearly distinguished in the lysate of washed platelets by Western blotting technique.

The indirect method measures 0.25 mg/mL AA-induced TXB_2_ generation in PRP diluted to 30 × 10^9^/L by PDP. The produced TXB_2_ was separated from AA and from other interfering substances by sequential solid phase extraction [11]. In the final eluted sample TXB_2_ concentration was measured by competitive immunoassay (Assay Designs, Ann Arbor, MI) and it was expressed as pg TXB_2_ produced by 10^6^ platelets.

As among the generally used routine laboratory tests used for the detection of aspirin effect AA-induced platelet aggregation, ATP secretion and the VN Asprin assay were proven to be the most reliable tests [12], we also used these assays to detect the effect of aspirin on patients with CAD being on secondary prevention. VN Aspirin Assay was performed according to the manufacturer’s instructions and the results were expressed as Aspirin Reaction Units (ARU). Platelet aggregation and secretion in platelet rich plasma was induced by 500 µg/mL AA (Helena, Gateshead, UK) and was monitored in Chrono-Log 700 lumiaggregometer (Chrono-Log, Havertown, PA) for 8 min. Prior to the experiments the platelet count was adjusted to 260 × 10^9^ platelet/L. Aggregation was expressed as the percentage of maximal change in light transmission (Δtransmission  %). ATP secretion was quantitated by bioluminescence method using luciferin-luciferase reagent (Biothema AB, Handen, Sweden). Maximal ATP secretion was expressed as μmol ATP/10^11^ platelets.

### Measurement of COX1 and COX2 mRNA

COX1 and COX2 mRNA expression was determined in the platelets of 56 patients randomly selected out of the 144 subjects with CAD. RNA was isolated from leukocyte depleted platelets as described earlier [19]. The integrity of RNA samples was shown by determining the GAPDH 3ʹ:5ʹ signal ratio [20], the absence of RNA contamination from white blood cells was proven by RT-qPCR analysis of each sample for CD15 and HLA-DQβ mRNAs [21]. Reverse transcription and RT-qPCR reaction were carried out as described by Zsóri et al. [19]. Briefly, First Strand cDNA Synthesis Kit (Roche, Mannheim, Germany) was used for reverse transcription. The reaction solution was incubated at 42 °C for 60 min, and then at 94 °C for 5 min. No-template control and no-reverse transcriptase controls included in each run showed negative results. The absence of contaminating DNA was also demonstrated by melting curve analysis. RT-qPCR reactions were carried out on LightCycler 480 (Roche) in duplicates using SYBR Green I Master (Roche). PCRs were set up in a final volume of 20 µL consisting 10 µL Master Mix (2× concentration), 5 µL of cDNA template derived from reverse-transcribed RNA and 300 nM primers for COX1 and 400 nM primers for COX2. The primer sequences were tccatgttggtggactatgg (forward), gtggtggtccatgttcctg (reverse) for COX1, and cttcacgcatcagtttttcaag (forward), tcaccgtaaatatgatttaagtccac (reverse) for COX2. ACTB, GNAS and HDGF have been established as the most stable reference genes for the normalization of platelet mRNA expression in coronary artery disease; all three were amplified and used in the calculations [19]. The amplification program was: heating for 10 min at 95 °C, followed by 40 cycles of 10 s at 95 °C, 30 s at 60 °C and 1 s at 72 °C. Melting curve analysis was performed between 66 and 95 °C in 0.11°C/s increments with 5 acquisitions/ °C. C_T_ values, corresponding to the number of cycles at which the fluorescence signal exceeds a threshold value, were used for the relative gene quantification based on the method of Livak and Schmittgen [22]. ΔC_T,COX1_ and ΔC_T,COX2_ were established against each of the three reference genes and the mean ΔΔC_T,COX1,COX2_ values were used for the calculation of COX1 mRNA:COX2 mRNA ratios.

## Results

### The acetylation of COX-1 by aspirin in patients with CAD

**Fig. 1:**
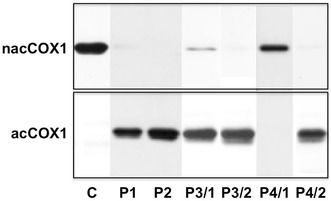
Detection of acetylated COX1 (acCOX1, *lower panel*) and non-acetylated COX1 (nacCOX1, *upper panel*) in platelet lysate of non-treated controls (C) and patients on aspirin treatment (P1–P4). Monoclonal antibodies specific to the acetylated and non-acetylated forms of COX1 were used in Western blotting detection system. In patients P3 and P4 non-compliance was presumed and the test was repeated after a 2 weeks period of compliance (P3/2 and P4/2). Below the band representing COX-1 another low intensity band cross-reacting with both antibodies is also apparent. It very likely represents COX-1 isoform 2, a 37 amino acids shorter transcript variant (http://www.ncbi.nlm.nih.gov/nuccore/18104968)

The characteristics of patients enrolled in the study are shown in Table 1. In the platelet lysate of 138 patients only acetylated COX1 was detected by Western blotting. These patients were represented by samples P1 and P2 in Fig. 1. Previously it has been shown that the method is highly sensitive and could detect as low as 2.5 % of the total platelet COX1 in non-acetylated form [11], i.e. more than 97.5 % of platelet COX-1 was acetylated as the consequence of long term aspirin treatment. It is to be noted that in platelets from these treated patients no nacCOX1 could be detected, which contradicts an earlier finding showing that only one monomer of the active dimeric enzyme is acetylated by aspirin [23]. In the case of six patients different amounts of non-acetylated COX1 remained in the platelet lysate. Lanes P3/1 and P4/1 show the two extremes; in sample P3/1 only a small amount of COX1 remained non-acetylated, while in a single sample (P4/1) no acetylation occurred. As noncompliance was assumed, these patients were contacted and their attention was drawn to the danger of not taking or irregularly taking the drug. After an additional two weeks the tests were repeated and full effectiveness of aspirin was demonstrated by the absence of non-acetylated COX1 and the presence of acetylated COX1 in the platelet lysate (see representative results on lanes P3/2 and P4/2, Fig. 1). According to sample size calculations the lack of aspirin resistance in all of the 144 patients indicated that aspirin is effective in more than 98 % of patients with stable coronary artery disease (confidence interval 95 %). It is to be noted that aspirin was fully effective in diabetic patients and also in smokers.

**Table 1:** Characteristics of patient population

Age (years)	64 ± 10 (42–85)
Male gender	67 %
BMI (kg/m^2^)	30 (26–33; 17–51)
Current smoker	10 %
Ever smoker	61 %
Diabetes mellitus	24 %
Triglyceride (mmol/L)	1.54 (1.1–2.1; 0.6–6.5)
Total cholesterol (mmol/L)	4.6 (3.8–5.4; 2.2–8.8)
HDL-C (mmol/L)	1.2 (1.1–1.5; 0.7–2.5)
LDL-C (mmol/L)	2.5 (2.0–3.3; 1.0–6.1)
Platelets (g/L)	237 ± 58 (100–432)
Fibrinogen (g/L)	3.7 (3.3–4.2; 2.0–7.1)
CRP (mg/L)	2.1 (0.9–4.3; 0.5–20.5)
Serum creatinine (µmol/L)	79 (68–90; 49–179)
GFR (mL/min/1.73 m^2^)	86 (70–91; 23–91)

Mean ± SD and total range in parenthesis are shown in the case of parameters with normal distribution and median with interquartile range and total range are given for parameters with non-normal distribution

*BMI* body mass index, *HDL*-*C* high-density lipoprotein cholesterol, *LDL*-*C* low-density lipoprotein cholesterol, *CRP* C-reactive protein, *GFR* glomerular filtration rate

### The effect of aspirin on arachidonic acid induced TXB_2_ production

**Fig. 2:**
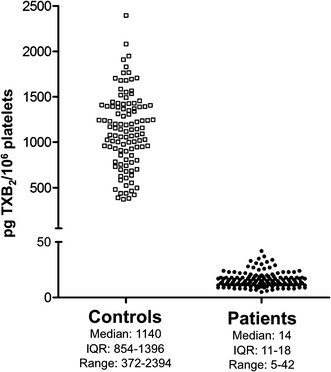
Arachidonic acid induced TXB_2_ generation of platelets from controls (*open square*) and from patients with coronary artery disease being on long-term aspirin treatment (*filled circle*). *IQR* interquartile range

Figure 2 demonstrates the AA induced TXB_2_ production of platelets in controls and in CAD patients being on long-term aspirin treatment. Aspirin drastically decreased the TXB_2_ generation, the median TXB_2_ production was only 1.2 % of that measured in the control group. Please, note that the y axis on the plot is broken and in the low range the scale is different. Here six patients also had higher TXB_2_ generation in the range of 43–835 pg TXB_2_/10^6^ platelets, but at the second occasion their value returned to the range of the remaining 138 patients. In Fig. 2 their samples are represented only with the results of the second determinations.

### The effect of aspirin on arachidonic acid induced platelet activation

**Fig. 3:**
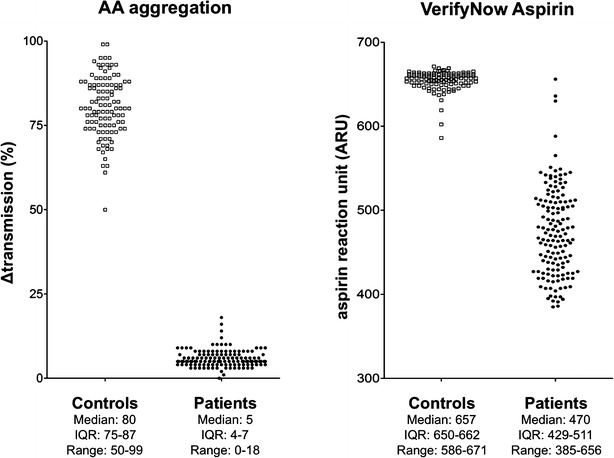
The results of arachidonic acid induced platelet aggregation and VerifyNow Aspirin Assay in controls (*open square*) and in patients with coronary artery disease being on long-term aspirin treatment (*filled circle*). *IQR* interquartile range

In accordance with the considerably decreased TXB_2_ production, the extent of AA induced aggregation of platelets from aspirin treated patients was uniformly very low and no overlap between controls and patients was observed (Fig. 3). The median value of AA induced ATP release in PRP was 1.1 μmol ATP/10^11^ platelets (IQR: 0.9–1.4) in the control group, while in the PRP of all aspirin treated patients the amount of released ATP was below the limit of detection. In the case of VerifyNow Aspirin assay four ARU values from the patients group (2.8 %) overlapped with the total range of ARU values of the control group (Fig. 3). For this assay the manufacturer recommends 550 ARU as the cut off value for aspirin effect, however according to the results of the control group a cut off value of 585 ARU seems to be more appropriate. The reason for the slight difference between the other assays and the VerifyNow Aspirin Assay, the latter also uses AA as agonist, is not clear. Maybe the detection system used in the VerifyNow Aspirin Assay (aggregation of fibrinogen coated beads) is responsible for the few outliers in the patient’s group.

It is to be noted that 24 % of the study group were diabetic patients, the CRP level was above 5 mg/L in 21 % of the patients and in spite of the clinical condition 10 % of them was unable to quit smoking. In none of these patients was impaired aspirin effect measured by any of the methods used in the study.

### The effect of aspirin on platelets with COX2 mRNA expression

**Fig. 4:**
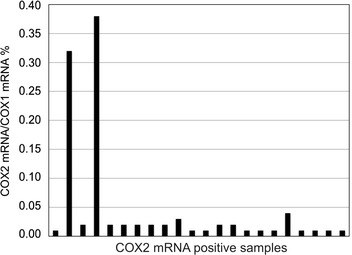
COX2:COX1 mRNA ratios in the platelets of patients with detectable COX2 mRNA

Out of the 56 patients investigated for the expression of COX2 mRNA only in 22 cases was the amount above the limit of detection (Fig. 4). Even in these cases the relative amount of COX2 mRNA was less than 0.4 % of COX1 mRNA and with the exception of three patients this value was less than 0.05 %. None of the patients expressing COX2 mRNA in platelets showed aspirin resistance.

## Discussion

So far, the direct measurement of COX1 acetylation in healthy volunteers taking 100 mg aspirin has been carried out in two studies using different methodologies. Our former study measured nacCOX1 and acCOX1 by Western blotting technique using specific monoclonal antibodies [11], in another study platelet proteins were separated by nuPAGE then, in-gel enzymatic digestion was carried out and the COX1 peptide carrying the Ser529 residue was determined by mass spectrometry [13]. One hundred and eight and twenty-four individuals were enrolled in the studies, respectively and in none of the cases was true aspirin resistance revealed. It is to be noted that using AA-induced platelet aggregation and serum TXB_2_ measurement Grosser et al. [24] also failed to detect specific phenotype of true aspirin resistance among 400 healthy volunteers. As in patients with CAD the occurrence of aspirin resistance has not been explored by detecting the acetylation of COX1 in platelets, our method developed as part of basic research was translated to address this question in clinical setting. In this study none of the 144 patients enrolled in the study showed impaired acetylation of platelet COX1, TXB_2_ generation, AA-induced platelet aggregation and ATP release were profoundly inhibited by aspirin in all patients. Neither diabetes nor smoking influenced the effectiveness of aspirin and inflammatory condition shown by elevated CRP also failed to influence COX1 acetylation. This finding suggests that in patients with coronary artery disease, just like in healthy individuals aspirin resistance, if it exists at all, is a rarity.

Several factors have been suggested to influence the effect of aspirin in pathological conditions. Increased platelet turnover was presumed to release newly formed platelets with nacCOX1 into the circulation and produce enough TXA_2_ to activate platelets, even platelets with acCOX1 [14, 15, 25, 26]. This problem was thought particularly important in the case of enteric-coated aspirin administered in a single daily dose. However, important component of the inhibitory effect of aspirin occurs in the presystemic circulation [13, 27]. Furthermore, COX1 of megakaryocytes is acetylated by aspirin and platelets released from megakaryocytes contain acCOX1 [28]. Furthermore, the finding of a persistent level of acCOX1 throughout a 7 days interval of treatment with once daily 100 mg enteric-coated aspirin provides direct evidence that acetylated platelets entered into the circulation during this time-frame [13]. Our present finding demonstrating that patients on long-term aspirin contain more than 97.5 % COX1 in acetylated form also support the cumulative nature of platelet COX1 inhibition upon repeated daily dosing [29]. The reports on the increased level of COX1 acetylation 24 h after the sixth daily dose of aspirin as compared to the level measured 24 h after a single aspirin dose also indicate cumulative saturable acetylation of platelet COX1 [11, 13].

COX2, an isoenzyme of COX1, is also sensitive to the inhibitory effect of aspirin [8, 30]. However a much higher dose of aspirin, well above the range reached during the prophylactic low dose aspirin therapy, is needed to acetylate the respective serine residue (in this case Ser516) [16]. This enzyme is present in megakaryocytes, it is expressed in young platelets [31] and its up-regulation has been described in diabetes mellitus and inflammatory conditions [17, 32, 33]. It has been proposed that COX2 expression in platelets might result in the suppression of aspirin effect. However, Riondino et al. [34] demonstrated on 100 patients being on chronic aspirin treatment by immunoblot analysis that COX2 could be detected only in 46 % of patients and its amount was markedly lower than that of COX1. By using COX2 inhibitor CAY10404 and aspirin they also demonstrated that COX2 dependent TXA_2_ production is less than 2 %. Our results are in line with the latter observations. We found detectable amount of platelet COX2 mRNA only in 39 % of patients with stable coronary artery disease. Even in those cases in which COX2 mRNA was detected, its amount was incomparably lower than that of COX1 mRNA and platelets from these patients produced a very low amount of TXB2 comparable to the rest of the aspirin treated patients. These findings do not support the role of COX2 expressed in platelets in the diminished response to low dose aspirin therapy.

In the light of the above findings it is a question if aspirin therapy should be monitored at all. In theory, mutations in the COX1 gene may result in a situation in which aspirin is unable to acetylate the enzyme, however such a situation has not been reported. The results suggest that the effect of aspirin does not need to be controlled in patients with coronary artery disease, unless non-compliance or drug interference is suspected. The main reason for the ineffectiveness of aspirin as detected by laboratory tests is non-compliance [35–37], testing of patients on aspirin therapy could provide information on their compliance. Non-steroid anti-inflammatory drugs may interfere with the effect of aspirin [38–40] and insufficient aspirin effect measured by adequate laboratory tests could draw the attention to such drug interference. It is to be emphasized that for the above purposes only methods specific to the effect of COX1 acetylation should be used. Among the routinely used methods these include AA induced platelet aggregation and secretion, the VerifyNow Aspirin Assay and the measurement of serum TXB_2_ [41]. This statement is valid only for aspirin monotherapy, its combination with P2Y12 receptor inhibitors (dual antiplatelet therapy) might influence the test results [42]. A limitation of the study is the patient population that comprised of individuals with stable coronary disease. It is to be seen if the results could be extended to patients with acute coronary syndrome.

## Conclusions

The acetylation of platelet COX1 was tested in 144 patients with stable coronary artery disease. A direct method based on monoclonal antibodies against nacCOX1 and acCOX1 failed to detect aspirin resistance among the patients. Similar results were obtained with the measurement of AA induced TXB2 production of platelets, with AA induced platelet aggregation and ATP secretion and the VerifyNow Aspirin assay. These methods are useful in detecting non-compliance and very likely also drug interference. COX2 mRNA was expressed in 39 % of 56 investigated patients in very low amount, but it was not related to impaired aspirin effect.
